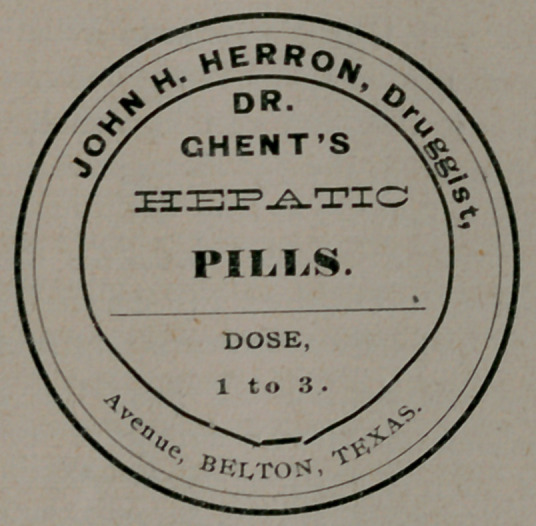# Dr. Ghent’s Hepatic Pills

**Published:** 1890-12

**Authors:** 


### EDITORIAL DEPARTMENT.

F. E. DANIEL, M. D., EDITOR AND PUBLISHER.

THIS JOURNAL, by unanimous vote of the members, was made the Official Organ of the Austin District Medical Society.

COLLABORATORS:

*Dr. R. M. Swearingen, Austin.*
*Prof. B. E. Hadra, M. D., Galveston.*
*Prof. Geo. Cupples, M. D., San Antonio.*
*Dr. T. C. Osborn, Cleburne, Texas.*
*Dr E. J. Doering, Chicago.*
*Dr. E. J. Beall, Fort Worth.*
*Dr Odo Betz, Germany.*
*Prof. W. B. Rogers, M. D., Memphis.*
*Dr. J. W. McLaughlin, Austin.*
*Dr. T. J. Tyner, Austin.*
*Prof. J. F. Y. Paine, M. D., Galveston.*
*Dr. R. H. L. Bibb, Mexico.*
*Dr. T. J. Bennett, Austin.*
*Dr. Bat Smith, Wharton.*
*Dr. E. Meierhof, New York.*
*L. H. Luce, M. D., West Tisbury, Mass.*

The above is a *fac simile* of the printed label on a box of pills dispensed by a Belton druggist,—whether on prescription or not our deponent sayeth not, but he very pertinently asks "if it is ethical for an ex-President of the Texas State Medical Association to have any hepatic pills, (dose 1 to 3)?

By reference to the Transactions of the State Medical Association for 1887 it will be seen that a member was *expelled* by the Judicial Council—not for making "hepatic pills"—but for *being associated in practice with one who did!* What they would have done with the "one who did"—(had he been a member) make and dispense pills called by his own name, can only be conjectured.

In the case of an ex-President, a zealous advocate of the suppression of Quackery, and one who is on record as having compiled facts to show the great prevalence of quackery in Texas—and who has written often and much on medical ethics—it certainly does not look well, to say the least, to see his name attached to a box of "hepatic pills" (dose 1 to 3)—in a proprietary connection. To have his druggist to make and dispense a pill bearing such a label, or even to permit him to do so, seems to us clearly a violation of the spirit, if not the letter of the code, and is glaringly inconsistent with the pretentions and utterances of the distinguished Henry Clay Ghent,—the erst-while "legislator-loud" in the demands for "regulating the practice" and "suppressing quackery" in Texas. Whether or not the fact that his druggist manufactures and keeps in stock (with his permission, of course) "Dr. Ghent's Hepatic Pills"—(dose 1 to 3)—along with "Dr. McLean's Liver Pills," etc., is actionable, and should subject him to the same rule by which the member above referred to was measured, the JOURNAL prefers not to express an opinion; but—it is a favorite saying with us—a crude expression of an inborn sense of equity—that "sauce for the goose *ought to be* sauce for the gander." Or, does the mantle of the Presidential office—like that of "charity"—cover a multitude of faults?—or, to paraphrase the old English law—can the President (of the State Medical Association) do no wrong?

In the light of such revelations as the above, is it any wonder that the common people cannot discriminate between the regular and the irregular in medicine? Buying a box each of "Dr. McLean's Liver Pills" and "Dr. Ghent's Hepatic Pills," can farmer Hayseed be expected to appreciate the vast gulf that separates Dr. G. from Dr. McL.? or see any difference, except that supposed—in his "untutored mind" to exist—in the words "liver" and "hepatic"? And is not even *this* taking an advantage of his untutored mind, to say "hepatic" instead of plain "liver,"—(a familiar word to the country folks)—and *make him believe*,—as Weller, Sr., said of Sam's "walentine"—"it means more?" Put it plain, "Dr. Ghent's Liver Pills, and Dr. McLean's

Liver Pills, and no farmer on earth could see any difference between them, nor between the positions the two doctors bear relatively to the people and the profession.

The honor of the Presidency in medical associations is supposed to be bestowed on the most worthy—the most examplary members. They ought to be as Mentors, or beacon lights to their followers. They should set the example in their walk and conduct, to lead to the highest and purest lives, morally, socially and professionally. They should in their daily walk exemplify the spirit of the code, and thus incite to higher aims. If our leaders descend to questionable methods and practices—is it fair to visit summary punishment in the lesser lights who follow their example? What respect for the code could a student of Dr. Ghent have—in knowledge of his "hepatic pills" being on sale along with Dr. Tutt's and Dr. Carter's at the village drug store?

## Figures and Tables

**Figure f1:**